# ﻿Morphological and ultrastructural studies of the internal reproductive systems of two deltocephaline leafhoppers, *Nephotettixcincticeps* and *Deltocephalusvulgaris* (Hemiptera, Cicadellidae, Deltocephalinae)

**DOI:** 10.3897/zookeys.1182.111297

**Published:** 2023-10-20

**Authors:** Jiarui Chen, Jing Zhang, Wei Liu, Bismillah Shah, Christopher H. Dietrich, Yani Duan

**Affiliations:** 1 Anhui Province Key Laboratory of Integrated Pest Management on Crops, Key Laboratory of Biology and Sustainable Management of Plant Diseases and Pests of Anhui Higher Education Institutes, School of Plant Protection, Anhui Agricultural University, Hefei, Anhui 230036, China Anhui Agricultural University Hefei China; 2 Jingxian Agricultural and Rural Bureau, Xuancheng, Anhui 242500, China Jingxian Agricultural and Rural Bureau Xuancheng China; 3 Department of Forestry Protection, School of Forestry and Biotechnology, Zhejiang A&F University, 666 Wusu Street, Linan, Hangzhou, Zhejiang 311300, China Zhejiang A&F University Hangzhou China; 4 Illinois Natural History Survey, Prairie Research Institute, University of Illinois, Champaign, IL 61820, USA University of Illinoi Champaign United States of America

**Keywords:** Auchenorrhyncha, comparative study, Memberacoidea, transmission electron microscope

## Abstract

Insects have highly variable reproductive systems, reflecting a diversity of reproductive strategies and adaptations. Such variation has been widely used to classify and estimate phylogenetic relationships. Here, the morphology and ultrastructure of the internal reproductive systems of two leafhopper species are described and illustrated, using both light and transmission electron microscopy, and representing two tribes of Deltocephalinae: in Chiasmini, *Nephotettixcincticeps* (Uhler, 1896), and in Deltocephalini, *Deltocephalusvulgaris* (Dash & Viraktamath, 1998). Tables comparing the morphology of male and female internal reproductive structures of these studied species are provided and indicate that the main differences are in the relative shapes, sizes, and colors of these structures. The overall structure and organization, including details of the ultrastructure, of these two leafhopper species’ male and female internal reproductive systems are very similar to those of previously studied leafhoppers. The main differences observed among species include the number of testicular follicles, the relative position of seminal vesicles and the degree of development of the accessory glands in the male, the number of ovaries, and the shape and color of the vagina and spermatheca in the female.

## ﻿Introduction

Insects are the dominant component of terrestrial biodiversity. Their ability to survive across complex and varied natural environments is closely related to their strong reproductive capacity. The structures of the insect reproductive system are complex and varied, reflecting different reproductive strategies (Song 2011). The reproductive system of insects consists of multiple organs in the abdominal cavity that work together to produce sperm or ova and facilitate copulation. A study of the insect reproductive system is needed to improve the understanding of insect reproductive potential and oviposition mode, which may ultimately facilitate pest management (Lei and Rong 2003). Comparative study of the reproductive structures of different insects also increases our understanding of the evolution and phylogeny of insects.

Despite leafhoppers’ high diversity and economic importance and the widespread use of the external sclerotized structures of their genitalia for taxonomy and phylogenetics, their internal reproductive organs remain little studied. Helms (1968) described in detail the morphology of the leafhopper *Empoascafabae* (Harris, 1841) male reproductive system. The adult male consists of two testes (each composed of four follicles), vasa deferentia with seminal vesicles, paired accessory glands, paired ejaculatory ducts, and a median ejaculatory bulb. Moreover, Mishra (1979) concluded in the comparison of male leafhoppers *Cofanaspectra* (Distant, 1908), *C.unimaculata* (Signoret, 1854), and *Amritodusatkinsoni* (Lethierry, 1889) that the number of testicular follicles is different, and the corresponding accessory glands also differ in shape. Similarly, Tsai and Perrier (1996) studied the morphology of male reproductive systems of the two deltocephaline leafhoppers representing different tribes, *Dalbulusmaidis* (DeLong, 1923) and *Graminellanigrifrons* (Forbes, 1885). They found that the males of these species have two lateral testes (each with six follicles). A pair of accessory glands arise at the posterior of each seminal vesicle and open into a lateral ejaculatory duct. Hayashi and Kamimura (2002) conducted a specific study on the leafhopper *Bothrogoniaferruginea* Fabricius, 1787 and found that during the process from production to entry into the female body, male sperm first transported to the female’s bursa copulatrix through a spermatophore. After successful mating, the sperm enters the spermatheca for fertilization. At this stage, the sperm-binding material (trypsin degradable proteins) and the spermatophore disappear in the bursa and an enlarged portion of the genital duct. They suggested that females could incorporate proteinaceous material derived from male spermatophores and/or sperm-binding material into their oocytes. Tian et al. (2006) compared the male reproductive systems of 39 species of Cicadellidae and focused on spermatogenesis, classifying the testes into two types according to the presence or absence of the sheath. Their results revealed that the testicular follicles of Cicadellidae are spherical, while testicular follicles in Heteroptera are lamellar and sheathed. Su et al. (2014) compared the ultrastructure of the male reproductive systems of *Psammotettixstriatus* (Linnaeus, 1758) and *Exitianusindicus* (Distant, 1908) (Deltocephalinae) and described the morphology and ultrastructure of the sperm of these two species. The sperm of both species have a 9+9+2 axonemal pattern, as in other Auchenorrhyncha, and provide a reference for the comparative study and phylogeny of other groups. Vitale et al. (2015) analyzed the morphology of the male reproductive system of *Balcluthabrevis* (Lindberg, 1954). Their results showed that secretory activity occurring mainly in the lateral ejaculatory ducts and the accessory glands. The ultrastructural features of the seminal vesicle differed from those of the lateral ejaculatory duct, suggesting that these structures play distinct roles in the organization of the sperm bundles. Recently, Zhang et al. (2016) found differences in the organizational structure of the male reproductive systems of the closely related cicadelline leafhoppers *Cicadellaviridis* (Linnaeus, 1758) and *Kollapaulula* (Walker, 1858).

Similarly, Helms (1968) found that the female reproductive system of *E.fabae* (Harris, 1841) consists of two ovaries (each composed of four telotrophic ovarioles), lateral oviducts, a median oviduct, a spermatheca, a genital chamber with a pair of spermatozoal pouches, and a median accessory gland. Additionally, Cheung (1994, 1995) studied the female reproductive system of *Euscelidiumvariegatus* Kirschbaum, 1858; it was clear that the ovary is endotrophic, and the main source of nutrition for growth and development is also completed by trophoblasts. Studies on the ultrastructure of the cicada reproductive system have found ovarian sheaths and tracheae on the periphery of the ovarian tubes, which are called perimetrium. Similarly, Tsai and Perrier (1996) also compared the morphology of the female reproductive systems of *D.maidis* (DeLong, 1923) and *G.nigrifrons* (Forbes, 1885) and found that ovaries of these species contain six ovarioles, each with six follicles. In *G.nigrifrons* each ovariole usually contains only one egg within the last follicle, whereas the ovarioles of *D.maidis* usually contain two eggs. The ovarioles open into the lateral oviduct, common oviduct, and the vagina. Besides, Hummel et al. (2006) studied the development of the ovaries in *Homalodiscavitripennis* (Germar, 1821) and found that a single ovary is composed of 10 ovarioles, and the development of an ovary occurred in seven stages. Recently, Pappalardo et al. (2016) studied the ultrastructure of the female reproductive system of *Balcluthabrevis* (Lindberg, 1954). Their results showed that the female reproductive system has a morphological configuration comparable to most species of Auchenorrhyncha.

These previous studies indicate that characteristics of the reproductive system may provide a basis for phylogenetic analysis and classification. However, the available data are scattered, and studies have focused on different reproductive characteristics. Thus, additional comparative analyses are needed.

## ﻿Materials and methods

### ﻿Source of specimens

Specimens of deltocephaline leafhopper *Nephotettixcincticeps* were collected in mid-June 2020 and *Deltocephalusvulgaris* in early September 2020 using light-trap and net-sweeping techniques. The collection sites were concentrated in the urban area of Hefei in Anhui Province. Individual adult leafhoppers were collected into tubes, classified, identified, and placed into insect cages to reside and feed on relevant hosts for 3–5 days before being processed for anatomical study. The samples *N.cincticeps* contained five males and two females, while *D.vulgaris* contained five males and nine females. We used specimens without well-developed eggs in ovarioles for drawings and descriptions of females.

### ﻿Light microscopy

Fresh adult leafhoppers were placed in a −36 °C freezer for 5–10 min. The leafhopper’s abdomen was then immediately dissected under a light microscope (Motic, K-700HS). The abdominal epidermis was carefully removed with a dissecting needle to reveal the complete internal reproductive system of the leafhopper and transferred into a new concave slide with glycerin. It was then observed and photographed under a stereoscope (Nikon, SMZ1500). Photographs were edited using Adobe Photoshop CS6.

### ﻿Transmission electron microscope

Fresh adult samples were dried for 15 min before the dissection of the internal reproductive system. Samples were fixed in 2.5% glutaraldehyde in a 0.1 M phosphate-buffered saline (PBS) (pH 7.2) and washed several times in the same phosphate-buffered saline. The samples were then fixed with 1% osmium tetroxide for 1–2 h. Later, the samples were serially dehydrated with 30%, 50%, 70%, 80%, 90% and 95% ethanol solutions for 15 min each, then treated with 100% ethanol for 20 min. After dehydration, all samples were treated with pure acetone for 20 min and then with a pure embedding agent for 24 h. Infiltrated samples were embedded by heating them at 70 °C for 24 h to obtain an embedded sample block. The embedded block was then sectioned in an ultra-microtome to obtain sections of 100 nm which could be observed under a transmission electron microscope (Hitachi, HT-7700). Micrographs of each species were obtained from a single male and a single female.

### ﻿Morphological terminology

The morphological terminology used here mainly follows Tsai and Perrier (1996).

## ﻿Results

### ﻿Morphological observation of the internal reproductive systems

*Nephotettixcincticeps*

**Figure 1.**
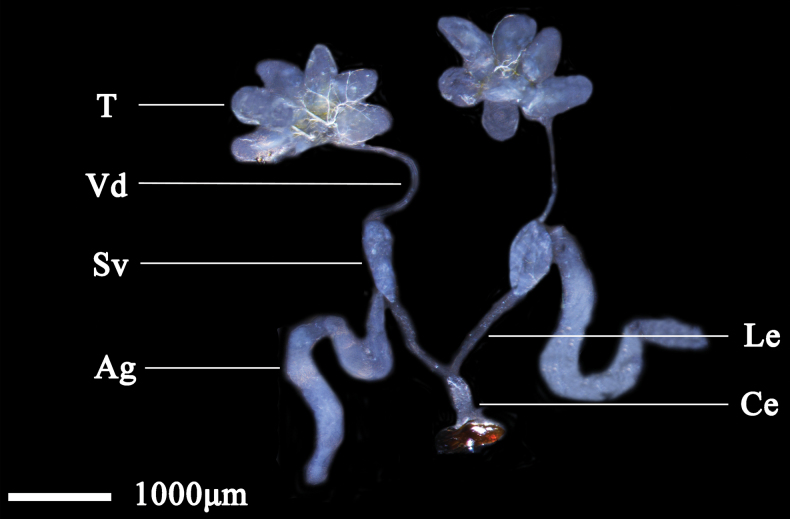
Male internal reproductive system of *N.cincticeps* (Uhler, 1896). Abbreviations: T – testis, Vd – vas deferens, Sv – seminal vesicle, Le – lateral ejaculatory duct, Ag – accessory gland, Ce – common ejaculatory duct.

The male internal reproductive system of *N.cincticeps* consists of a pair of testes, a pair of vasa deferentia, a pair of seminal vesicles, a pair of accessory glands, a pair of lateral ejaculatory ducts, and a single common ejaculatory duct. The testes consist of six testicular follicles of similar size and shape, which are droplet-shaped and pale blue, and their distal ends are connected to the slender vasa deferentia. The seminal vesicle is oval, outward spreading, and pale blue (same as the testicular follicles). The accessory glands are well developed, with a long, tubular structure and bilateral symmetry. The distal end of the seminal vesicle is contracted and joined to the lateral ejaculatory duct, a slender, tubular structure. The distal ends converge and expand into the common ejaculatory duct, which is straight and connected with the external genitalia (Fig. 1).

**Figure 2.**
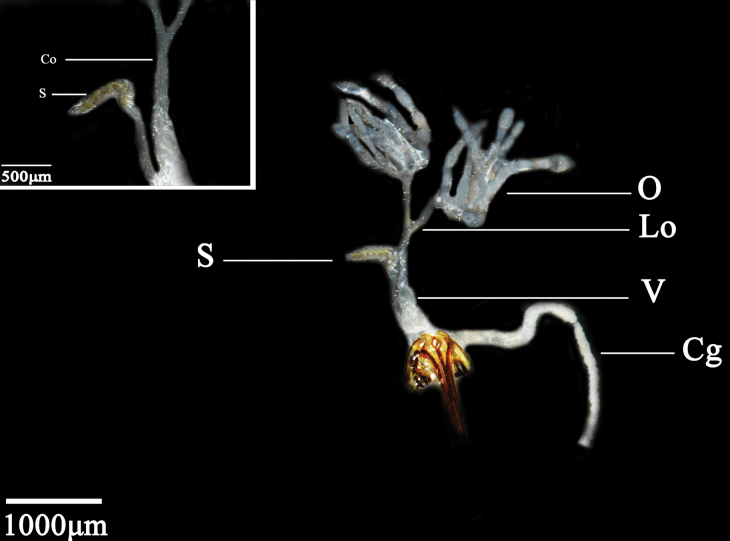
Female internal reproductive system of *N.cincticeps* (Uhler, 1896). Abbreviations: O – ovary, Lo – lateral oviduct, S – spermatheca, V – vagina, Cg – colleterial gland, Co – common oviduct.

The female internal reproductive system comprises a pair of ovaries, two lateral oviducts, a common oviduct, a colleterial gland, a vagina, and a spermatheca. A pair of accessory glands may also be present, but their degree of development varies according to the age and physiological state of the individual female (Hummel et al. 2006). The ovary is composed of six ovarioles of similar shape and size. The individual ovarioles are milky white, tubular, rounded at the top, and confluent at the distal ends to meet the lateral oviduct. The lateral oviducts are slender, tubular structures whose two distal ends fuse to converge with the common oviduct. The anterior part of the spermatheca is a slender, tubular structure. The termination of the common oviducts adheres to the vagina. The colleterial gland has a developed, tubular structure. The spermatheca is yellowish white and digitate in appearance (Fig. 2).

*Deltocephalusvulgaris*

**Figure 3.**
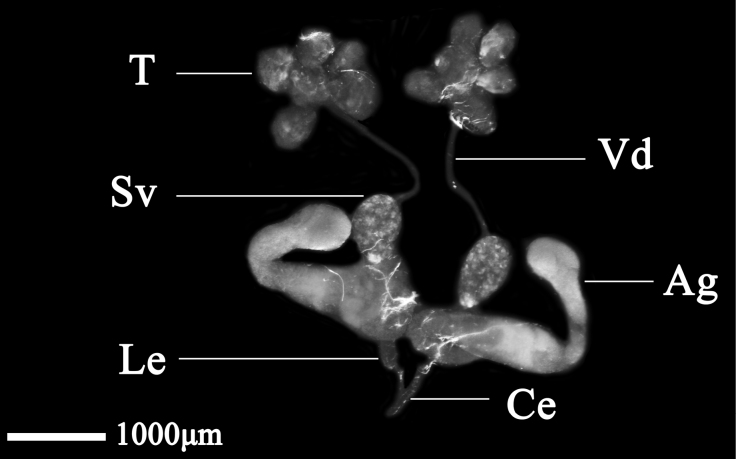
Male internal reproductive system of *D.vulgaris* (Dash & Viraktamath, 1998). Abbreviations: T – testis, Vd – vas deferens, Sv – seminal vesicle, Ag – accessory gland, Le – lateral ejaculatory duct, Ce – common ejaculatory duct.

The male internal reproductive system of *D.vulgaris* consists of a pair of testes, a pair of vasa deferentia, a pair of seminal vesicles, a pair of accessory glands, a pair of lateral ejaculatory ducts, and a single common ejaculatory duct. The testes are composed of five independent testicular follicles, which are pale blue with obvious bright spots, resemble a series of water droplets, and are connected to the vasa deferentia. The vasa deferentia are linear, slender, and connected to the seminal vesicle. The whole seminal vesicle is pale blue, translucent, and ovoid. The accessory glands are developed, the anterior segment is protuberant, the middle segment is contracted, the distal end is rod-like, the whole is milky white, and both sides are symmetrical. The lateral ejaculatory sac joins the common ejaculatory duct, which connects with the external genitalia (Fig. 3).

**Figure 4.**
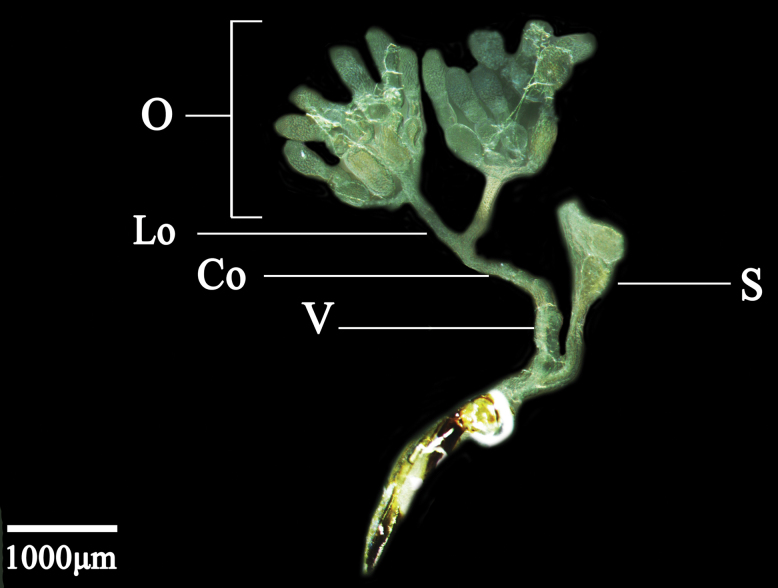
Female internal reproductive system of *D.vulgaris* (Dash & Viraktamath, 1998). Abbreviations: O – ovary, Lo – lateral oviduct, Co – common oviduct, S – spermatheca, V – vagina.

The female internal reproductive system consists of a pair of ovaries, lateral oviducts, a common oviduct, a colleterial gland, a vagina, and a spermatheca. The ovaries on both sides are symmetrically unfolded in a “Y” shape. Each ovary is made up of six ovarioles of similar shape and size. Individual ovarioles are milky white tubules, rounded at the top, and confluent at the distal ends to meet the lateral oviduct. The lateral oviduct is slender, thin, closed at both distal ends, and converges with the common oviduct. The distal end of the common oviduct adheres to the vagina. The spermatheca is light yellow and shaped like an irregular cone (Fig. 4).

### ﻿Ultrastructure of the internal reproductive systems

#### ﻿Ultrastructure of the male internal reproductive system of *N.cincticeps*

Testis

**Figure 5.**
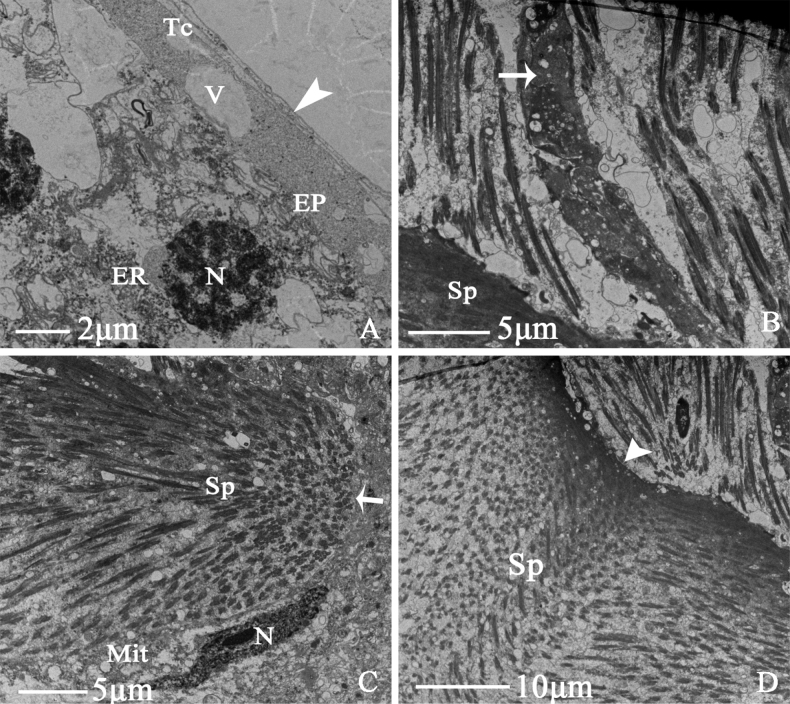
Ultrastructure of testicular follicle of *N.cincticeps* (Uhler, 1896) **A, B** cross-section of testicular follicle, showing (Tc) tracheole, (V) vesicle, (ER) endoplasmic reticulum, (EP) epithelium, (N) epithelial cell nucleus, (Sp) spermatid, (TM) the triangular arrowhead indicates testicular follicle membrane, (BM) the long arrow indicates thick basal membrane **C, D** showing (Sp) spermatid, (Mit) numerous mitochondria, (N) epithelial cell nucleus, (BL) the long arrow indicates basal lamina, (BM) the triangular arrowhead indicates thick basal membrane.

The testis comprises six droplet-shaped testicular follicles of similar size and shape. The testis is symmetrical, without a sheath, and the surface is pale blue. The testicular follicles comprise a follicle membrane, muscular sheath, epithelium, and lumen with sperm at different developmental stages. There are tracheoles between the testicular follicle membrane and the muscle sheath and vesicles in the epithelium. Many endoplasmic reticula surround epithelial cell nucleus. Many sperm gather in the testicular follicles, and a thick basal membrane is present (Fig. 5A, B). During the growth and development of the sperm, the sperm bundle is formed and is free to the edge of the basal membrane, at the same time, the phenomenon of partition appears (Fig. 5C, D).

##### Seminal vesicle

**Figure 6.**
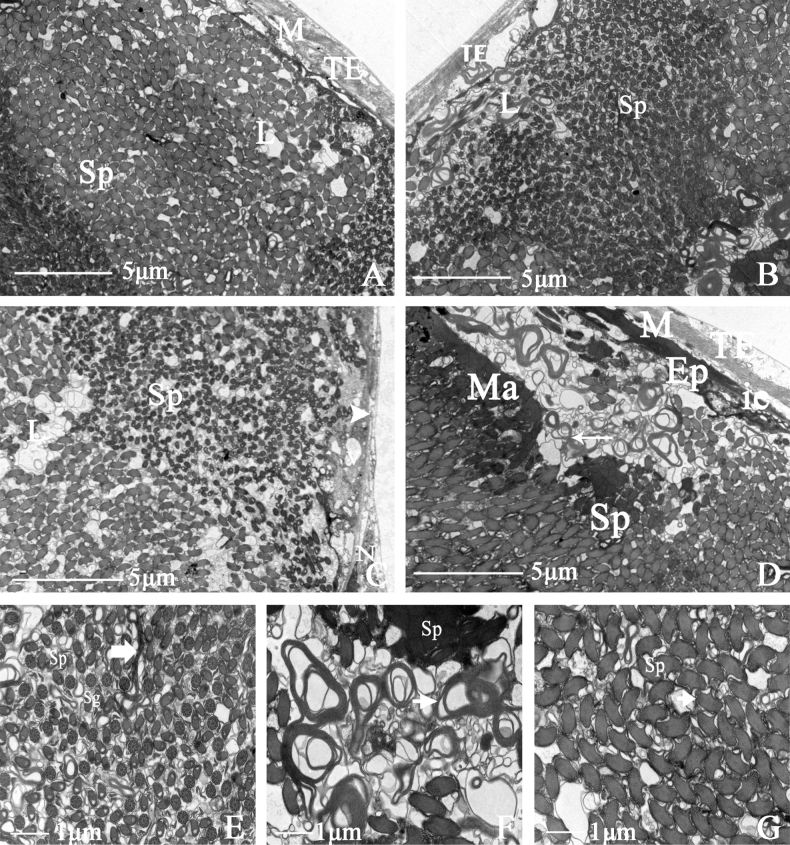
Ultrastructure of seminal vesicle of *N.cincticeps* (Uhler, 1896) **A, B** cross-section of seminal vesicle, showing (TE) tunica external, (M) muscle sheath, (Sp) sperm, (L) lumen **C** showing (L) lumen, (Sp) sperm **D** showing (TE) tunica external, (M) muscle sheath, (EP) epithelium, (ie) intercellular spaces, (Sp) sperm, (Ma) head of sperm embedded in the homogenous matrix in lumen **E** showing (Sp) sperm and (Sg) secretory granules in lumen, accompanied by plasma membrane to gap (arrow) **F** the arrow indicates lamellar bodies **G** cross-section of thumbtack nuclei of sperm.

The edge of the seminal vesicle is clear, with starlike spots, almost oval, and pale blue. The tunica external tightly wraps the seminal vesicle, and the overall structure comprises four parts: tunica external, muscular sheath, epithelium, and lumen. There are some differences in sperm at different developmental stages (Fig. 6A, B). A long, narrow intercellular space exists between the epithelium and the muscular sheath. The lumen contains much sperm, and their heads are inserted into the matrix to form sperm bundles (Fig. 6C, D). Sperm and secretory cells appear simultaneously, with distinct plasma membrane spacing and lamellar bodies appearing in clumps in the lumen. In the cross-section of the seminal vesicle, the thumbtack-shaped nuclei and surrounding microtubules are visible (Fig. 6E–G).

##### Accessory glands

**Figure 7.**
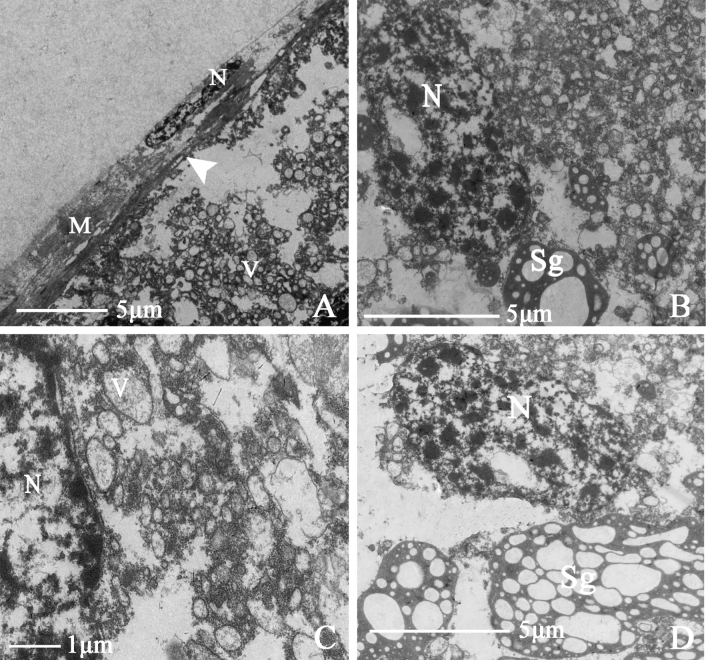
Ultrastructure of male accessory gland of *N.cincticeps* (Uhler, 1896) **A** cross-section of accessory gland, showing (M) muscle sheath, (N) epithelial cell nucleus, (BL) the white-arrowhead indicates basal lamina **B–D** showing (N) epithelial cell nucleus, (V) vesicle, (Sg) secretory granules.

The accessory glands of *N.cincticeps* are long, tubular structures with bilateral symmetry. Their structure is relatively simple, consisting of a muscular sheath, epithelium, and basal lamina from the outside to the inside (Fig. 7A). The central part of the tube has a large amount of secretory material. Secretory granules with variable margins and vesicles surround epithelial nuclei. Multiple secretory granules gather to form a secretory center surrounded by mitochondria (Fig. 7B–D).

#### ﻿Ultrastructure of the female internal reproductive system of *N.cincticeps*

##### Vagina

**Figure 8.**
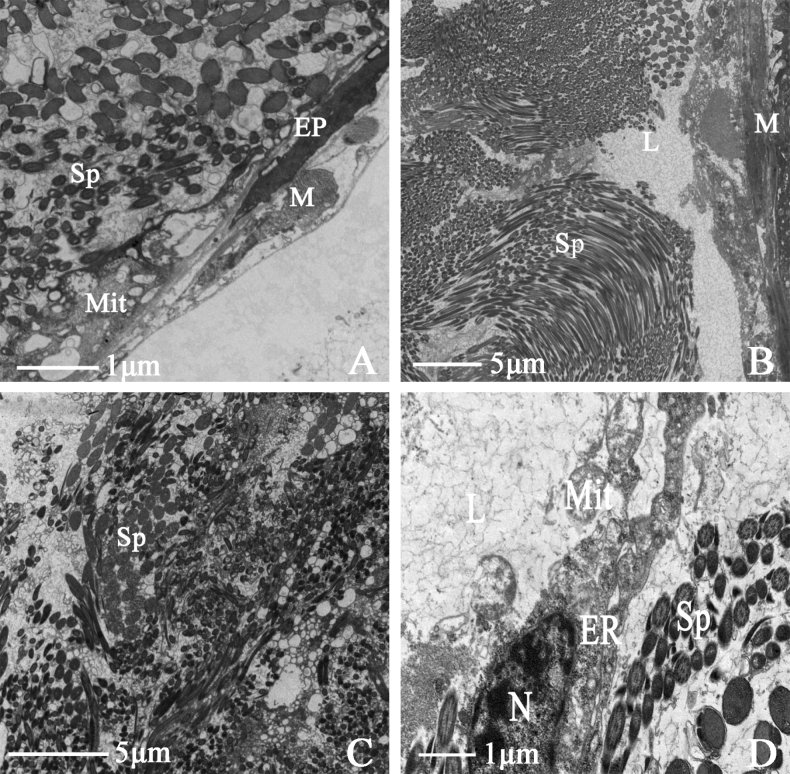
Ultrastructure of vagina of *N.cincticeps* (Uhler, 1896) **A, B** cross-section of vagina, showing (M) muscle sheath, (EP) epithelium, (Mit) mitochondria, (Sp) sperm, (L) lumen **C, D** showing (N) epithelial cell nucleus, (ER) endoplasmic reticulum, (L) lumen, (Sp) sperm, (Mit) mitochondria.

The vagina of *N.cincticeps* is a simple, short, thick tubular structure. Transmission electron microscopy shows that it is composed of a muscular sheath, epithelium, and lumen. There are abundant mitochondria at the junction between the muscular sheath and lumen. The endoplasmic reticulum surrounds the epithelial cell nucleus. The core of the illustrated specimen is occupied by much sperm (Fig. 8A, B). Sperm swim in the lumen in the direction of the matrix (Fig. 8C, D).

##### Spermatheca

**Figure 9.**
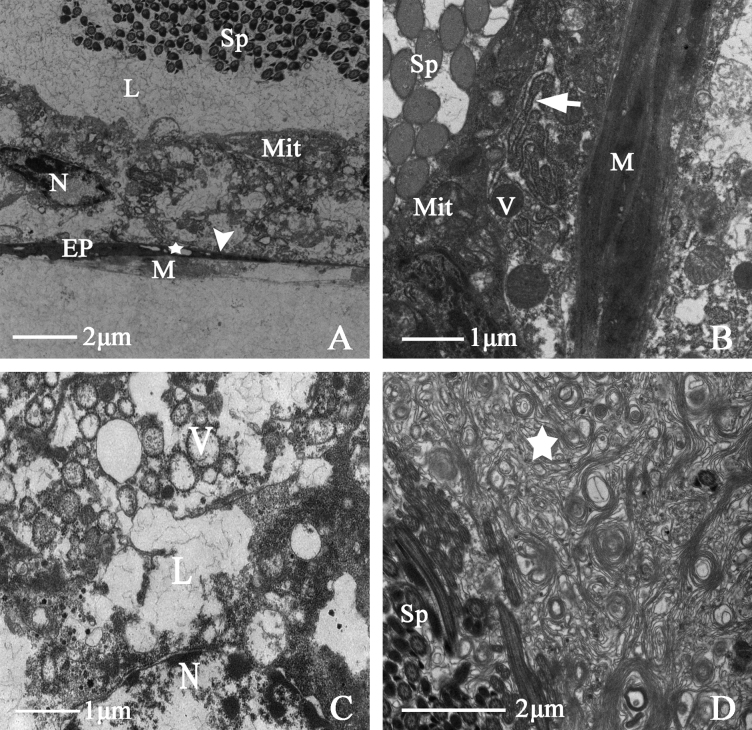
Ultrastructure of spermatheca of *N.cincticeps* (Uhler, 1896) **A** cross-section of spermatheca, showing (M) muscle sheath, (EP) epithelium, (L) lumen, (N) epithelial cell nucleus, (Sp) sperm, (BL) black triangular arrowhead indicates basal lamina **B, C** showing (M) muscle sheath, (V) vesicles, (Mit) mitochondria, (Sp) sperm, (L) lumen, (if) black arrowhead indicates infolding **D** showing (Sp) sperm.

The spermatheca base is slender and tubular, the distal end is enlarged to about 90° and bent outward, and the whole structure is pale yellow. Under transmission electron microscopy, the spermatheca is shown to be composed of a muscular sheath, epithelium, and lumen. The spermatheca has a hollow lumen filled with many sperm. Muscle texture is clearly visible in the muscular sheath, and a few tracheoles are observed at the junction with the epithelium (Fig. 9A). Infolding is present at the interval between the epithelium and the basal lamina. Numerous mitochondria and vesicles surround epithelial cell nucleus (Fig. 9B, C). Sperm swim in the matrix, which is covered by abundant lamellar bodies (Fig. 9D).

#### ﻿Ultrastructure of the male internal reproductive system of *D.vulgaris*

##### Testis

**Figure 10.**
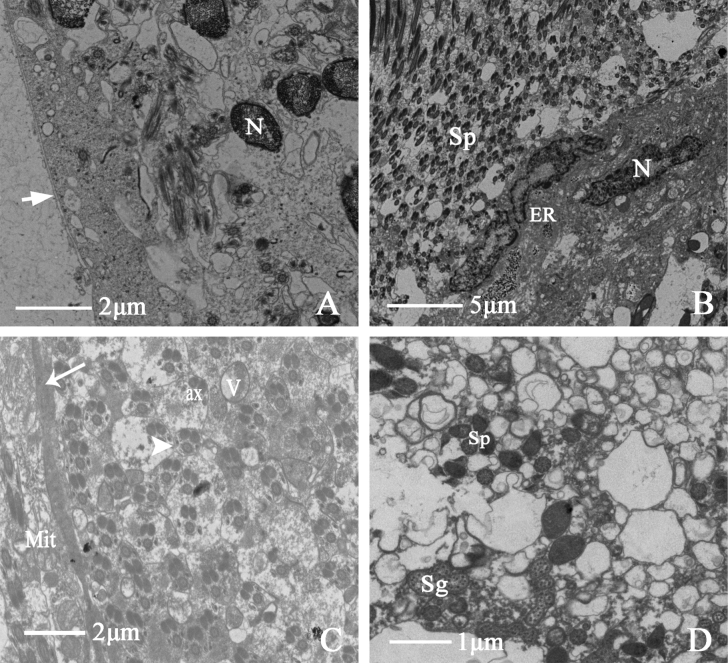
Ultrastructure of testicular follicle of *D.vulgaris* (Dash & Viraktamath, 1998) **A** cross-section of testicular follicle, showing (TM) testicular follicle membrane, (N) epithelial cell nucleus, (TM) the arrow indicates testicular follicle membrane **B** showing (Sp) spermatid, (ER) endoplasmic reticulum, (N) epithelial cell nucleus **C** showing (ax) axoneme, (V) vesicle, (Mit) mitochondria, (BM) the long arrow indicates thick basal membrane, (Sj) the triangular arrowhead indicates septate junction **D** showing (Sp) spermatid, (Sg) secretory granules.

The testis has a clear margin and comprises six droplet-shaped testicular follicles of similar size and shape. The testis is symmetrical, without a sheath, and the surface is pale blue. The testicular follicles comprise a testicular follicle membrane, muscular sheath, epithelium, and lumen with sperm at different developmental stages. Mitochondria surround epithelial cell nucleus. Microtubule material besides the epithelial cell nucleus converges at both distal ends (Fig. 10A). There is a clear dividing line between the sperm and epithelium. Spermatogenesis and development occur in the lumen (Fig. 10B). A single sperm has a cell boundary and a thick basal lamina. There are a few vesicles between spermatozoa (Fig. 10C). Secretory cells are close to sperm cells. Sperm have a typical 9+9+2 axonemal pattern (Fig. 10D).

##### Seminal vesicle

**Figure 11.**
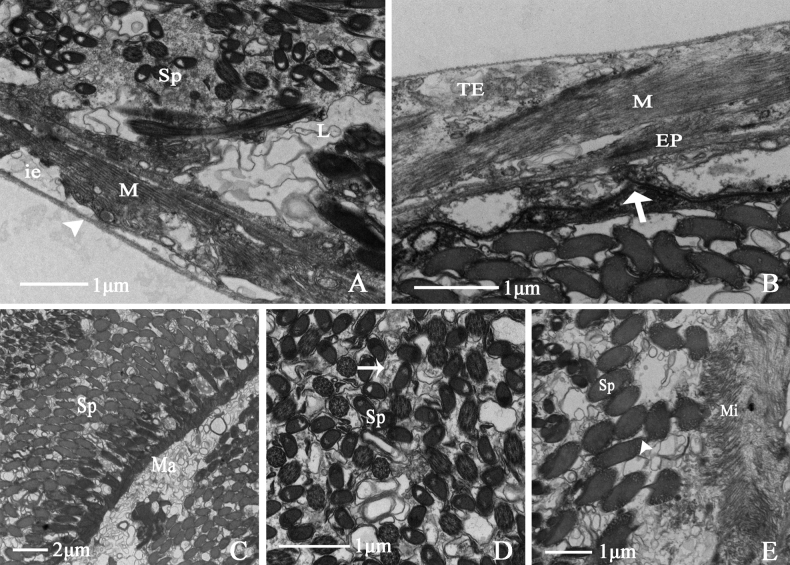
Ultrastructure of seminal vesicle of *D.vulgaris* (Dash & Viraktamath, 1998) **A, B** cross-section of seminal vesicle, showing (TE) tunica external, (M) muscle sheath, (EP) epithelium, (N) epithelial cell nucleus, (Sp) sperm, (L) lumen, (ie) intercellular spaces, (TE) the triangular arrowhead indicates tunica external, (BL) the long arrow indicates basal lamina **C–E** showing (Ma) head of sperm embedded in the homogenous matrix in lumen, (Sp) spermatid, (Sj) the arrow indicates a septate junction, (Mi) microvillus.

The edge of the seminal vesicle is clear, and the seminal vesicle has starlike spots, is almost oval, and pale blue. The structure of the seminal vesicle consists of four parts: the tunica external, muscular sheath, epithelium, and lumen. The lumen contains many sperm at different stages of development, the sperm heads are inserted into the homogenous matrix, and there is an intercellular septum between sperm. There is an intercellular space between the tunica external and the muscular sheath. The muscles of the muscular sheath are clearly textured and striated. The basal lamina at the edge of the epithelium folds inward (Fig. 11A, B). Microvilli in the epithelium increase the area of the secretory surfaces (Fig. 11C–E).

##### Accessory gland

**Figure 12.**
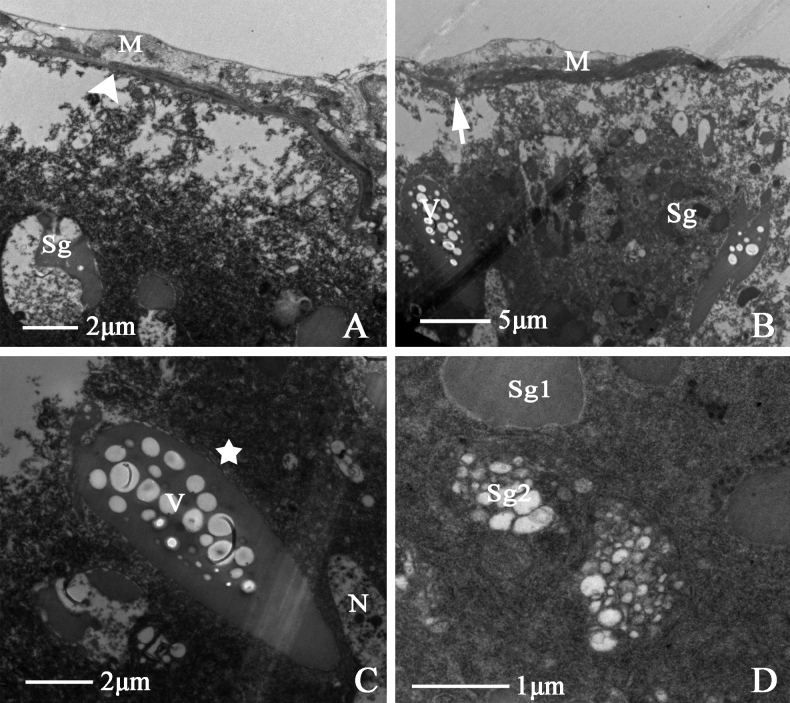
Ultrastructure of male accessory gland of *D.vulgaris* (Dash & Viraktamath, 1998) **A, B** cross-section of accessory gland showing (M) muscle sheath, (BL) triangular arrowhead indicates basal lamina, (if) the arrowhead indicates infolding, (Sg) secretory granules **C, D** showing (V) vesicle collection, (ER) the asterisk indicates endoplasmic reticulum, (N) epithelial cell nucleus, (Sg1) secretory granules 1, (Sg2) secretory granules 2.

The accessory gland of *D.vulgaris* is relatively large and with bilateral symmetry. The front segment of the accessory gland protrudes, while the middle segment is contracted; the distal end is rod-like, and the whole is milky white. Its structure comprises a muscular sheath, epithelium, basal lamina, and numerous secretory granules. A dark basal lamina is formed at the edge of the epithelium, accompanied by basal lamina folding (Fig. 12A, B). Around the secretory granules, many weakly electron-dense vesicles congregate in the secretory cell center, the margin is oval and surrounded by endoplasmic reticulum (Fig. 12C). Based on their morphology and composition, secretory granules can be divided into Sg1 and Sg2 (Fig. 12D).

#### ﻿Ultrastructure of the female internal reproductive system of *D.vulgaris*

##### Ovary

**Figure 13.**
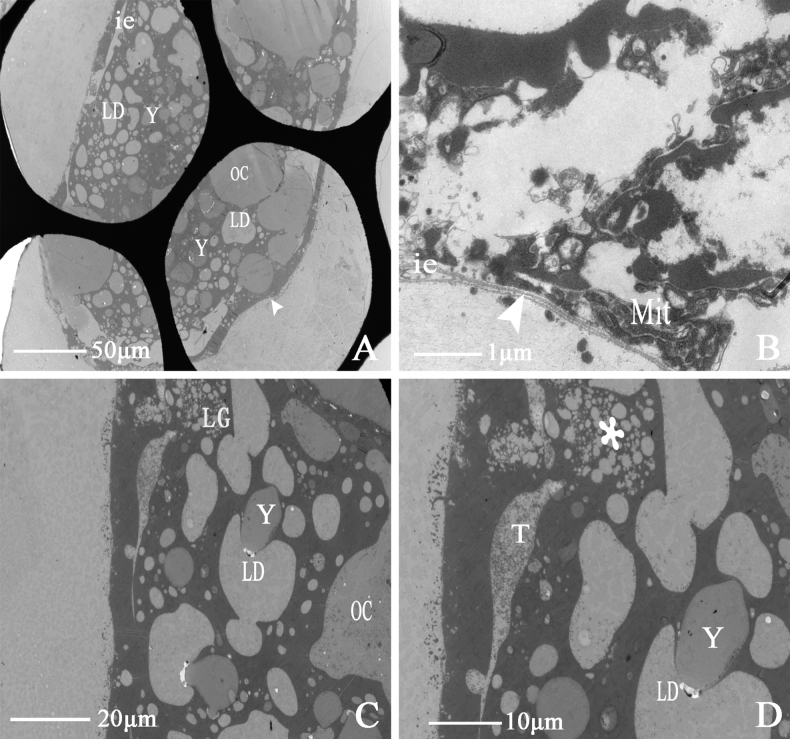
Ultrastructure of ovariole of *D.vulgaris* (Dash & Viraktamath, 1998) **A, B** cross-section of ovarioles, showing (OC) oocytes, (LD) lipid drop, (Y) yolk granule, (ie) intercellular spaces, (Mit) mitochondria, (BL) the arrow indicates basal lamina **C** showing (OC) oocytes, (LD) lipid drop, (Y) yolk granule, (LG) lipid granules **D** showing (T) trophocytes, (LD) lipid drop, (Y) yolk granule, (MB) the asterisk indicates a multivesicular body.

The ovaries of *D.vulgaris* are symmetrically expanded. A single ovariole is a milky-white tubular structure, rounded at the apex, with its distal end joining the lateral oviduct. At low magnification under transmission electron microscopy, the ovaries are oval and contain oocytes, fat droplets, and yolk granules. The margin of the basal lamina is clear, and the intercellular space is visible (Fig. 13A, B). Many yolk granules, fat droplets, and lipid granules surround the oocyte. Trophocytes vary in morphology (Fig. 13C). Yolk granules and fat droplets embed into each other, enlarging as the ovariole develops. There are multiple vesicles (Fig. 13D).

##### Vagina

**Figure 14.**
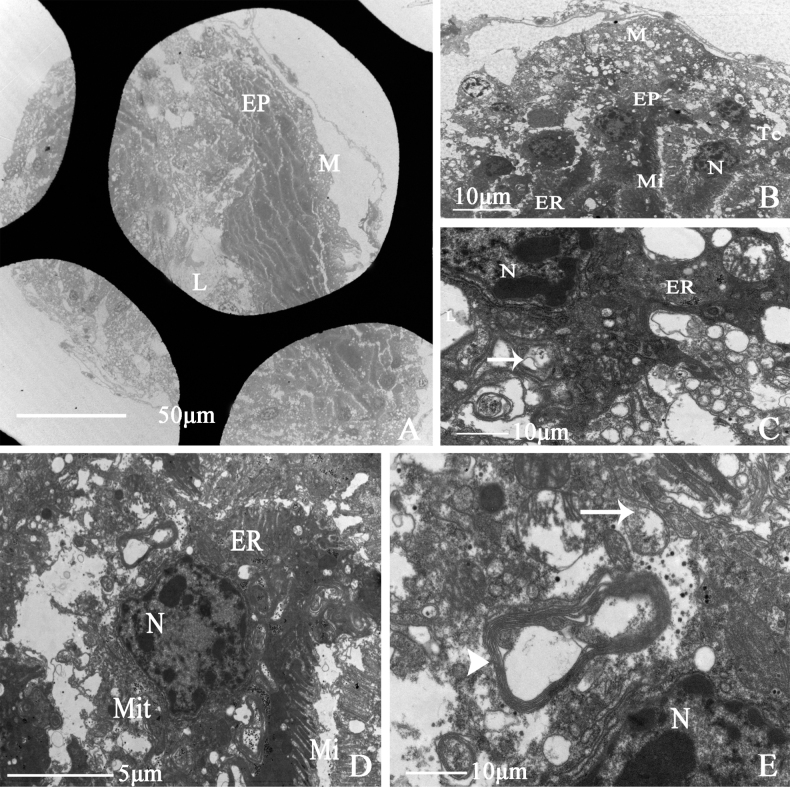
Ultrastructure of vagina of *D.vulgaris* (Dash & Viraktamath, 1998) **A** cross-section of vagina, showing (M) muscle sheath, (EP) epithelium, (L) lumen **B, C** showing (M) muscle sheath, (EP) epithelium, (ER) endoplasmic reticulum, (Mit) mitochondria, (Tc) tracheole, (Sj) the arrow indicates septate junction **D** showing (N) epithelial cell nucleus, (ER) endoplasmic reticulum, (Mit) mitochondria, (Mi) microvillus; (E) showing (N) epithelial cell nucleus, (Sj) the long arrow indicates the septate junction.

The vagina of *D.vulgaris* is a tubular structure and connects with the distal end of the common oviduct. Transmission electron microscopy shows that it is composed of a muscular sheath, epithelium, and lumen. The epithelium is thicker, mottled, and stretched into strips (Fig. 14A). Tracheoles are present at the junction of the muscular sheath (Fig. 14B). Epithelial nuclei are surrounded by abundant mitochondria and an endoplasmic reticulum. No sperm was observed in the vagina of the specimen illustrated here. Cells are separated by a curved intercellular septum (Fig. 14C). Microvilli are observed in the epithelium (Fig. 14D). There are scattered unidentified, black, granular materials in the epithelium, accompanied by lamellar bodies (Fig. 14E).

## ﻿Discussion

In this study, the overall morphology and ultrastructure of the male and female internal reproductive systems of two species of Deltocephalinae are described and illustrated for the first time. The overall composition and structure of the internal genitalia of these species are similar to those of other studied leafhoppers. Although we observed some differences between *N.cincticeps* and *D.vulgaris*, further study will be needed to determine whether such differences, e.g., in the color of the seminal vesicles of the males and degree of development of the spermatheca and accessory glands of the females, are consistent or whether they may reflect different ages or physiological stages of the studied individuals.

Comparing our observations to the few published observations of the male internal reproductive organs of other leafhopper species (Table 1), we note that, in contrast to the two species of Deltocephalinae studied here, in which males have the seminal vesicles well separated from each other, as in the megophthalmine leafhopper *Agalliaconstricta* (Gil-Fernandez & Black, 1965), another deltocephaline species, *B.brevis* (tribe Macrostelini) has the seminal vesicles joined to each other medially (Vitale et al. 2015). Two previously studied species of sharpshooters (subfamily Cicadellinae) also have paired seminal vesicles joined medially (Tian et al. 2006; Zhang et al. 2016). Vitale et al. (2015) also noted that the male accessory glands of *B.brevis* are divided into distinct proximal and distal sections distinguishable by color. Such sections are evident in the accessory glands of *K.paulula* but not in *C.viridis*, based on illustrations by Zhang et al. (2016), and we also did not observe distinct regions in the two species examined here. However, the distal section of the accessory gland of *D.vulgaris* is somewhat paler than the darker proximal section. In other respects, leafhoppers’ male internal reproductive structures appear to be highly conservative, although differences among species in the number of testicular follicles have been reported (Vitale et al. 2015), as observed among the two species studied here.

**Table 1.** Main features of male internal reproductive systems of Cicadellidae.

Subfamily	Tribe	Species	Testicular follicles	Seminal vesicle	Accessory gland	Lateral ejaculatory sac	Common ejaculatory duct	Reference
Cicadellinae	Cicadellini	*Bothrogoniaferruginea*	11–13, globular	2, oval, close integration	2, tubular, developed	Long, slender, tubular	Globular	Hayashi and Kamimura 2002
		*Cicadellaviridis*	6, globular	2, columnar, close integration	2, tubular, short, white	Long, slender, tubular	Globular	Zhang et al. 2016
		*Cofanaspectra*	3, oval	2, close integration	2, wide tubes	Long, straight	Expanded	Mishra 1979
		*Cofanaunimaculata*	5	2, close integration	2, wide tubes	Long, convoluted	Expanded	Mishra 1979
		*Kollapaulula*	5, globular	2, teardrop, close integration	2, tubular, long, peachblow	Long, slender, tubular	Globular	Zhang et al. 2016
Deltocephalinae	Chiasmini	*Exitianusindicus*	6, dacryoid, yellow	2, immediate, columnar, forsythia	2, tubular, short, white	Short, thick, straight	Sausage, white	Su et al. 2014
		*Nephotettixcincticeps*	6, teardrop, baby blue	2, expanded, oval, baby blue	2, tubular, developed, white, transparent	Long, slender, tubular	Tubular, white	Here examined
	Deltocephalini	*Deltocephalusvulgaris*	6, teardrop, baby blue	2, expanded, oval, baby blue	2, tubular, milky white	Short, slender, tubular	Tubular, white	Here examined
		*Graminellanigrifrons*	6, oval	2, elliptical, immediate	2, tubular, long, thick	Long, thin, straight	Elliptical, white	Tsai and Perrier 1996
	Macrostelini	*Balcluthabrevis*	6, oval	2, immediate, yellow	2, tubular, distal 2/3 yellow/brown, proximal 1/3 white/ opalescent	Long, thin, straight	Elliptical, white	Vitale et al. 2015
		*Dalbulusmaidis*	6, oval	2, elliptical, immediate	2, tubular, long, thick	Long, thin, straight	Elliptical, white	Tsai and Perrier 1996
	Paralimnini	*Psammotettixstriatus*	6, dacryoid, yellow	2, immediate, splayed, yellow	2, tubular, short, thick, white	Heliciform	Globular, white	Su et al. 2014
Eurymelinae	Idiocerini	*Amritodusatkinsoni*	6	2, separate	2, long, narrow, coiled	Short, straight	Expanded	Mishra 1979
Typhlocybinae	Empoascini	*Empoascafabae*	4	2, oval	2, tubular	Short, tubular	Globular	Helms 1968

Comparing our observations to the few published observations of the female internal reproductive organs of other leafhopper species (Table 2), we note that, among female leafhoppers, the most obvious differences among species in the internal reproductive structures seem to be the number of ovarioles. The two studied species each had six ovarioles per ovary, as in most previously studied leafhopper species. However, the number of ovarioles may be much larger and vary within a species, e.g., 8–10 in *H.vitripennis* (Hummel et al. 2006). The structure of individual ovarioles may also vary among species, which may reflect differences in fecundity (Tsai and Perrier 1996), although such variation also occurs within individuals at different stages of development and vitellogenesis (Hummel et al. 2006). Much more comparative study, including the variation among individuals of the same species at different stages of development, is needed to elucidate further the morphological variability of leafhoppers’ male and female internal reproductive structures.

**Table 2.** Main features of female internal reproductive systems of Cicadellidae.

Subfamily	Tribe	Species	Ovariole	Common oviduct	Colleterial gland	Vagina	Spermatheca	Reference
Cicadellidae	Cicadellini	*Bothrogoniaferruginea*	—	Short, tubular	—	Globular	Expand	Hayashi and Kamimura 2002
	Proconiini	*Homalodiscavitripennis*	10, perlitic, milky white	—	1, tubular	Globular	Bursiform, 4 compartments	Hummel et al. 2006
Deltocephalinae	Athysanini	*Euscelidiusvariegatus*	7	Tubular	1, tubular	Globular	—	Cheung 1994, 1995
	Chiasmini	*Nephotettixcincticeps*	6, tubular, milk white	Short, tubular	1, tubular, developed	Tubular, milk white	Crooked, tubular, faintly yellow	Here examined
	Deltocephalini	*Deltocephalusvulgaris*	6, tubular, milk white	Short, tubular	1, tubular, underdeveloped	Tubular, faint yellow	Triangular, faintly yellow	Here examined
		*Graminellanigrifrons*	6	—	1, tubular, underdeveloped	Globular, white	Globular, small	Tsai and Perrier 1996
	Macrostelini	*Balcluthabrevis*	6	Tubular, convoluted	—	Sac-shaped	Globular, small, brown	Pappalardo et al. 2016
		*Dalbulusmaidis*	6	—	1, tubular, underdeveloped	Globular, small	Globular, small	Tsai and Perrier 1996
Typhlocybinae	Empoascini	*Empoascafabae*	4	Tubular, convoluted	1, tubular	Club-shaped	Kidney-shaped	Helms 1968

Note: “—” information not reported.

## ﻿Conclusion

The overall structure and organization, including details of the ultrastructure, of the male and female reproductive systems of *Nephotettixcincticeps* (Uhler, 1896) and *Deltocephalusvulgaris* (Dash & Viraktamath, 1998) are very similar to those of previously studied leafhoppers. The main differences observed among species include the number of testicular follicles, the relative position of seminal vesicles, the degree of development of the accessory glands in the male, the number of ovarioles, and the shape and color of the vagina and spermatheca in the female. This suggests that, compared to the external genitalia, which highly varies among species and is often used in taxonomy, the internal reproductive structures of leafhoppers offer relatively few characters useful for classification and phylogenetics. Nevertheless, relatively few species have so far been studied in detail. Future work should focus on representatives of the many additional leafhopper subfamilies that have not yet been studied in detail.

## ﻿Additional information

### Author ORCIDs

Jiarui Chen https://orcid.org/0009-0002-7919-1972

Jing Zhang https://orcid.org/0000-0002-9354-786X

Wei Liu https://orcid.org/0009-0001-1727-0280

Bismillah Shah https://orcid.org/0000-0002-8407-8627

Christopher H. Dietrich https://orcid.org/0000-0003-4005-4305

Yani Duan https://orcid.org/0000-0002-5952-0778

### Data availability

All of the data that support the findings of this study are available in the main text.
